# Mechanical thrombectomy in a pediatric patient with antiphospholipid syndrome—a case report

**DOI:** 10.3389/fmed.2025.1530420

**Published:** 2025-04-11

**Authors:** Justyna Kowalczewska, Katarzyna Stanisławska, Joanna Rybacka-Mossakowska, Robert Juszkat, Sławomir Michalak

**Affiliations:** ^1^University Clinical Hospital in Poznan, Poznan University of Medical Sciences, Poznan, Poland; ^2^Department of General and Interventional Radiology, University Clinical Hospital in Poznan, Poznan University of Medical Sciences, Poznan, Poland; ^3^Department of Neurochemistry and Neuropathology, University Clinical Hospital in Poznan, Poznan University of Medical Sciences, Poznan, Poland; ^4^Department of Neurology, University Clinical Hospital in Poznan, Poznan University of Medical Sciences, Poznan, Poland; ^5^Department of General and Interventional Radiology, University Clinical Hospital in Poznan, Poznan University of Medical Sciences, Poznan, Poland; ^6^Department and Clinic of Neurosurgery and Neurotraumatology, University Clinical Hospital in Poznan, Poznan University of Medical Sciences, Poznan, Poland

**Keywords:** thrombectomy, pediatric stroke, antiphospholipid syndrome, acute ischemic stroke, stroke center

## Abstract

Stroke ranks among the top 10 most common causes of death in children. Recently, there has been a significant increase in the number of strokes in the pediatric population. Mechanical thrombectomy is an uncommon method for treating acute ischemic stroke (AIS) in children. This case report discusses a 13-year-old girl with a history of ischemic stroke for the past 3 months, obesity, hypertension, and antiphospholipid syndrome (APS, treated with rivaroxaban), who suffered another ischemic stroke and underwent mechanical thrombectomy with favorable clinical outcomes. Additionally, the patient was diagnosed with antithrombin III deficiency. It is necessary to identify risk factors for AIS in the pediatric population, such as thrombophilia or autoimmune diseases, such as antiphospholipid syndrome, and to develop guidelines for the use of thrombectomy in children. This method could reduce mortality, improve quality of life, prevent disability, and lower future medical costs.

## 1 Introduction

Mechanical thrombectomy is a widely recognized method for treating occlusions in large cerebral vessels (1). This procedure involves the use of endovascular tools to remove a blood clot from a blocked artery, restoring normal cerebral circulation (2). This operation can be performed using a stent, an aspiration catheter, and a combination of both (3). Many randomized clinical trials have confirmed its effectiveness (4). Mechanical thrombectomy is a well-established treatment for stroke in adults, but it is not commonly used in children (5–8). Due to insufficient data on the total number of thrombectomies performed in the pediatric stroke population and the lack of randomized clinical trials, the safety profile of this procedure remains unknown, making its use off-label (9–11). However, in 2015, the American Heart Association approved the use of thrombectomy in reasonable cases in the population aged below 18 years (12).

Strokes in children are much less common than in adults, with an incidence of approximately 3–25 per 100,000 in developed countries (10). Acute ischemic strokes (AISs) occur in the pediatric population at a rate of 1–2 cases per 100,000 individuals (13). AIS in adults is estimated to affect 236 cases per 100,000 individuals in the US population (14). Due to strokes in children being rare, diagnosing them may be delayed, resulting in disqualification from intravenous thrombolysis (IV tPA) due to exceeding the time window for this procedure (15, 16). In adults, the case of IV tPA lasts up to 4.5–9 h, while for thrombectomy, it extends to 6–24 h (11, 12, 17, 18). Therefore, thrombectomy can be performed in children who fall outside the time window for intravenous thrombolysis or who have contraindications (19).

In recent years, there has been an increase in the incidence of strokes among children worldwide, as well as in thrombectomy within the pediatric population (17, 20, 21). Stroke ranks among the top 10 most common causes of death in children (22). After an AIS, 3–6% of children die, 25% have recurrent strokes, and 70% are left with lifelong disabilities (19). The risk factors for stroke in children typically include arteriopathy (e.g., moyamoya), heart diseases, coagulation disorders (e.g., thrombophilia), acute head and neck disorders (e.g., meningitis), and acute and chronic systemic diseases (e.g., rheumatic diseases and autoimmune diseases) (10, 11, 13, 15). The incidence of strokes among children is rising due to the increasing prevalence of stroke risk factors in the pediatric population that were once typical of the adult population, such as cardiovascular risk factors, such as obesity, hypertension, and smoking (23–25).

This case report presents a girl with a history of antiphospholipid syndrome (APS) and ischemic stroke (3 months prior) who developed AIS symptoms for the second time and underwent mechanical thrombectomy.

## 2 Case report

**Figure 1:**
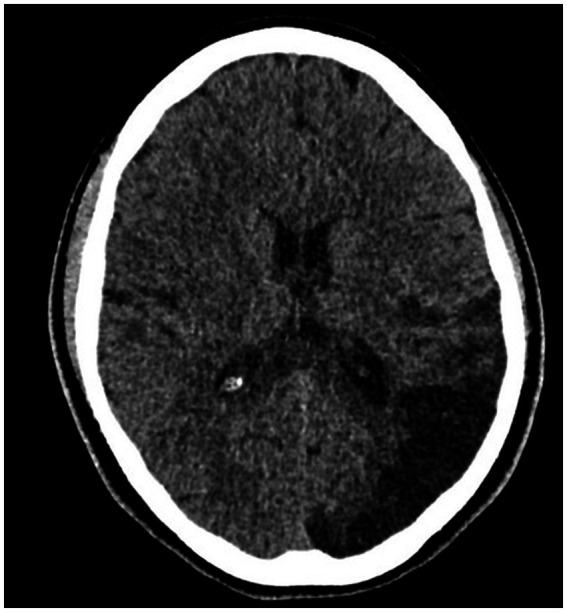
CT without contrast—ischemic changes in the left occipital lobe—the extent of vascularization of the terminal branches of the left posterior cerebral artery after the patient’s first stroke.

A 13-year-old girl was admitted to the stroke ward after being transferred from a children’s hospital due to an ischemic stroke. Three months earlier, an ischemic stroke with paresis of the right limbs had occurred (Figure 1). The patient presented with a history of antiphospholipid syndrome (positive anticardiolipin antibodies in IgM class and positive lupus anticoagulant) treated with rivaroxaban (diagnosed and treated after the first stroke), hypertension treated with ramipril, and obesity (weight: 84.5 kg, height: 167 cm, BMI: 30.3).

Upon admission, the patient was conscious, partially oriented, and turned the head and eyes to the right. The patient exhibited left hemianopia, left hemineglect, left hemihypesthesia, dysarthria, left central facial nerve palsy, flaccid plegia of the left upper limb, and paresis of the left lower limb, rated 3/5 on the Lovette scale. The patient received a total of 18 points on the Pediatric National Institutes of Health Stroke Scale (PedNIHSS). The blood pressure was 125/77 mm Hg, and the pulse was 73 beats per min. The laboratory results on admission were as follows: red blood cells (RBCs): 4.27 × 10^12^/L, hematocrit (HCT): 33.5%; platelets (PLTs): 255 × 10^9^/L; C-reactive protein (CRP): 10.5 mg/L; creatinine: 0.96 mg/dL: international normalized ratio (INR) 1.02; activated partial thromboplastine time (APTT): 30.9 s; D-dimer 1.2 μg/mL; antithrombin III: 19.19%; potassium: 4.4 mmol/L; sodium: 140 mmol/L.

**Figure 2:**
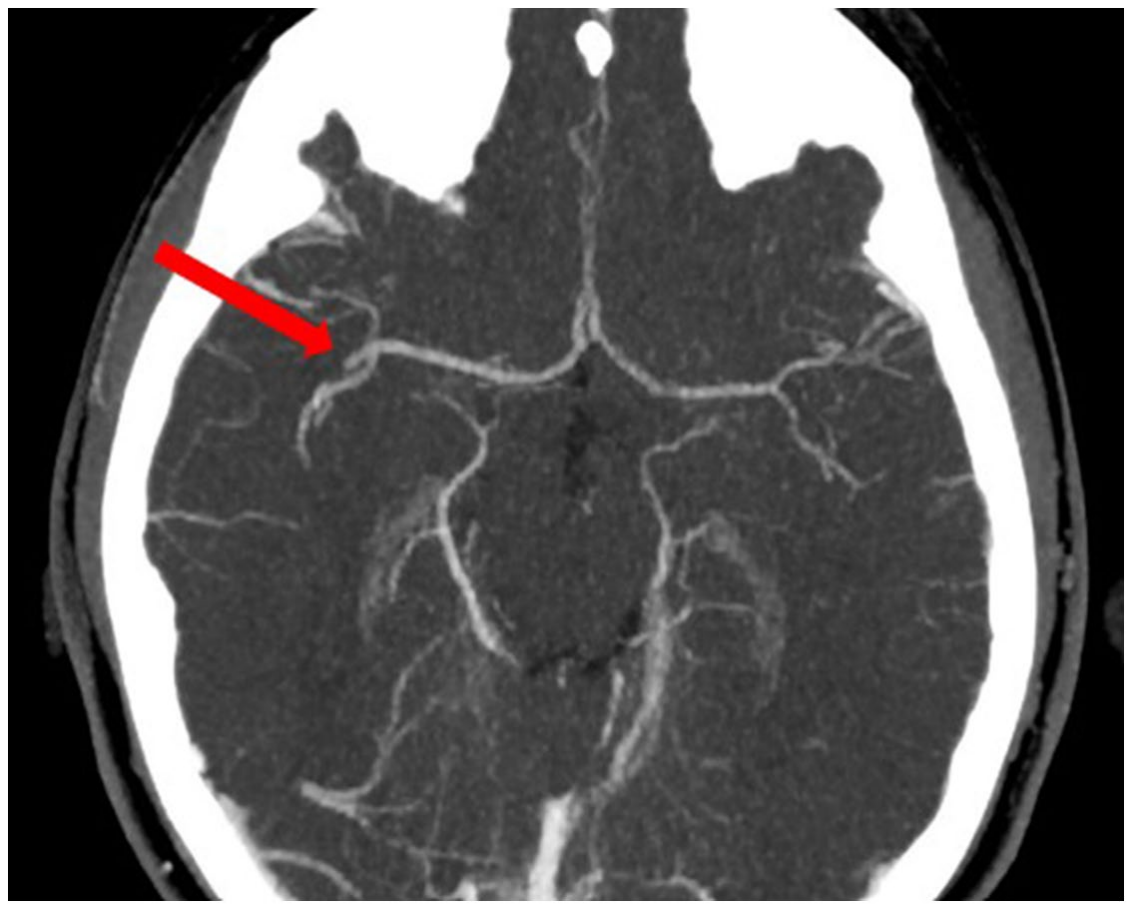
Angio-CT—occlusion of the M2 segment of the right middle cerebral artery before thrombectomy.

As the patient was taking rivaroxaban, she was disqualified from intravenous thrombolysis because contraindications to thrombolysis include the use of non-vitamin K antagonist anticoagulant (NOAC) within the last 48 h before the onset of symptoms (18). Angiography was performed through the right femoral artery. The angiography of the right carotid artery showed patency of the right internal carotid artery throughout its entire length, including its division (Figure 2). The right anterior cerebral artery was patent and morphologically normal. The right middle cerebral artery was patent in the M1 segment and in two of the three M2 segments. Until now, the patient had a Modified Rankin Scale (mRS) of 0 for neurologic disability and 9 points on the Alberta Stroke Program Early Computed Tomography Score (ASPECTS).

**Figure 3:**
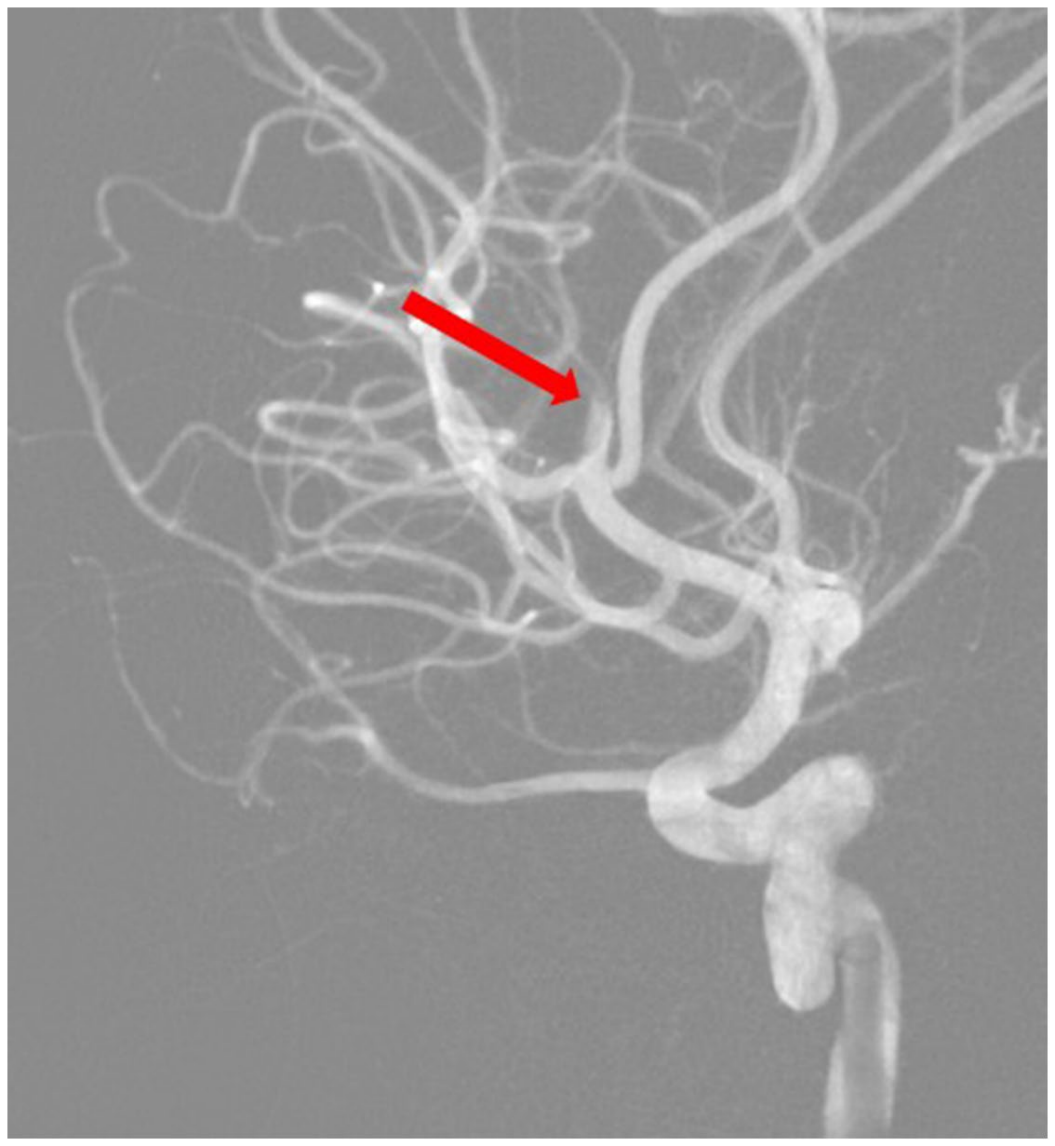
Digital subtraction angiography road map before thrombectomy.

**Figure 4:**
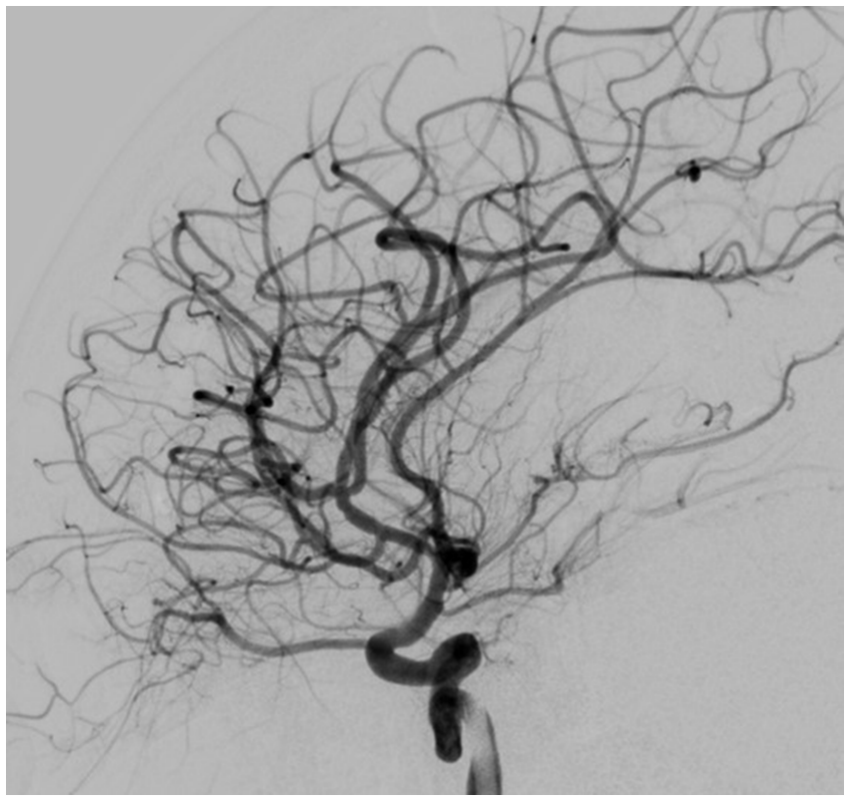
Digital subtraction angiography after thrombectomy.

Based on all signs and symptoms following a medical council, the patient was deemed eligible for mechanical thrombectomy. The need for the procedure was explained to the patient and her parents, and consent for the procedure was signed. The procedure was performed under general anesthesia; 3 h and 15 min after the onset of symptoms, a mechanical thrombectomy of the right middle cerebral artery was performed using the Penumbra aspiration system, with the guiding catheter Neuron MAX 088 (Penumbra, Alameda, CA, United States). Through the guiding catheter, two aspiration catheters were inserted—the smaller 4MAX with an inner diameter of 1.04 mm navigated with microguidewire Whisper LS 0.014 (Abbott, Plymouth, MA, United States) to aspirate from the M2 segment; after that, the second catheter, JET7, with an inner diameter of 1.83 mm, to remove the migrated thrombus from the M1 segment (both segments from Penumbra, Alameda, CA, United States). Complete patency of the right middle cerebral artery with its branches was obtained (Figures 3, 4). The procedure was performed without complications. Thrombolysis in Cerebral Ischemia Scale (TICI) scale was 0 before thrombectomy and 3 after.

The patient was transferred to the anesthesiology and intensive care ward of the children’s hospital for further treatment with recommendations, including performing a control computed tomography (CT) after 24 h and administering acetylsalicylic acid (ASA) and heparin in a prophylactic dose in the absence of hemorrhage. A control CT in the children’s hospital revealed complete patency of the right middle cerebral artery in the M2 segment; no intracranial hemorrhage was found. A larger hypodense area near the right insula cortex was visualized compared to the previous CT. After thrombectomy, the patient received 8 PedNIHSS points.

Following the procedure, the paresis of the left lower limb improved. As a result of further treatment and rehabilitation, the symmetry of the face improved, and the paresis of the left limb disappeared.

During her stay in the children’s hospital, she was suspected of antithrombin III deficiency, which additionally predisposed her to the occurrence of AIS. After treatment with ASA, low molecular weight heparin, and supplementing the deficiency of antithrombin III on the seventh day after the stroke, warfarin treatment was initiated under INR control. After obtaining the appropriate INR, heparin was discontinued. After being discharged from the hospital, the patient was ordered to continue physical rehabilitation and prescribed warfarin with target INR values ranging from 2.5 to 3. The patient did not have adequate treatment with vitamin K antagonists (VKA) before; she was treated with rivaroxaban; hence, the decision to treat with warfarin. We do not have complete data on the choice of rivaroxaban treatment in this case because the diagnosis of antiphospholipid syndrome (APS) and the initiation of therapy after the first stroke took place outside our country, and the patient’s documentation in this respect is incomplete. According to the Food and Drug Administration (FDA) and the European Medicines Agency (EMA), rivaroxaban is authorized for the treatment of venous thromboembolism (VTE) and for preventing VTE recurrence in children and adolescents under 18 years of age (26). Hassan and Motwani (27) describe a group of 64 patients in whom rivaroxaban was used to treat acute VTE. They assess its use in the pediatric population as safe, effective, and well-tolerated. In accordance with the European Alliance of Associations for Rheumatology (EULAR) guidelines for the management of antiphospholipid syndrome in adults, rivaroxaban should not be used in patients with triple antiphospholipid (aPL) antibody positivity due to the high risk of recurrent events. Direct oral anticoagulants (DOACs) could be considered only in patients unable to achieve a target INR despite good adherence to VKA or those with contraindications to VKA. Based on the current literature, there is no recommendation to use DOACs in patients with definite APS and arterial events due to the high risk of recurrent thrombosis (28). According to the systematic review by Pastori et al. (29), DOACs can be used in patients who are already on stable anticoagulation with a DOAC, are experiencing low-quality anticoagulation with warfarin, are unwilling or unable to undergo INR monitoring, or have contraindications or serious adverse events while on warfarin. In patients with venous APS with single or double positivity, the use of DOACs may be considered, but further research is necessary. Adult APS DOACs have been linked to a higher rate of recurrent thromboses in arterial thrombosis compared to warfarin, but this has not been observed in venous thrombosis (26). Bhoelan et al. (30) suggest that the use of DOACs in patients with venous thromboembolism (VTE) in hereditary antithrombin (AT) deficiency is not less effective in secondary prevention in this group of patients. A cohort study conducted in 2024 on the efficacy and safety of using DOACs in inherited antithrombin deficiency showed that at standard doses, DOACs are effective and relatively safe (31).

No further ischemic changes occurred in the patient’s follow-up 10 months after thrombectomy. In the angio-CT, the internal carotid artery showed no signs of dissection or stenosis.

## 3 Discussion

Antiphospholipid syndrome (APS) is a rare multisystem autoimmune disease in children characterized by the presence of aPL in the blood, including anticardiolipin antibodies, anti-beta-2-glycoprotein antibodies, and lupus anticoagulant. The most crucial goal of APS treatment is to prevent thrombotic episodes by using warfarin preceded by heparin therapy. It is also essential to prevent and treat other prothrombotic risk factors, such as obesity and hypertension. Eculizumab and sirolimus appear promising in treating APS, but so far, there is insufficient data regarding their use in children (32). APS affects approximately 3% of the pediatric population before the age of 15 and is more common in girls (33, 34). This patient was diagnosed with antiphospholipid syndrome at the age of 13 after her first ischemic stroke. Clinical manifestations of APS may include thrombotic and non-thrombotic events, pregnancy problems, neurological, dermatological, hematological, renal, and other manifestations (35). The most characteristic clinical picture of APS in the pediatric population is venous thrombosis, mainly of the lower limbs, and arterial thrombosis (approximately 32% of cases) causing ischemic stroke (79% of arterial thrombosis). Stroke in children with APS is more common than in adults (in Slovenian children with APS, stroke occurred in 26% of cases) (33, 36, 37). The risk of AIS in children with APS was found to be 6–8 times higher in studies from Israel and Canada (38, 39). The cause of death in patients with APS is often thrombotic events. For this reason, pediatric APS is a life-threatening disease with a high mortality rate and a significant reduction in patients’ quality of life (32). An even rarer issue is recurrent thrombosis in children with APS, which concerns the patient from this case report. Mayo Clinic researchers reported that AIS recurrence in children diagnosed with APS occurred in 59% of patients in the study group (of which 80% did not receive a therapeutic dose of anticoagulants). The average time between thrombotic events was 1.4 years (40). In this case report, AIS recurrence occurred during rivaroxaban therapy within 3 months from the first AIS. In a single-center study from New York, 10% of patients treated with rivaroxaban had a recurrence of arterial thrombosis; similar results were obtained by researchers from Italy, where 19% of APS patients treated with rivaroxaban had a recurrence compared to 3% treated with warfarin (41, 42). Recurrent thrombosis in pediatric patients with APS occurs in more than 50% of cases, prompting the establishment of effective anticoagulation regimens in this population and highlighting the importance of finding other effective treatments (43). It should also be noted that recurrent thrombosis in APS is more common in children than in adults (44). Cerebral ischemia can be caused by the occlusion of cerebral arteries, leading to AIS or TIA (transient ischemic attack). Both of these conditions are rare in the pediatric group; therefore, the occurrence of arterial thrombosis in a child may suggest a diagnosis of APS in a particular patient (44, 45).

The management of stroke in adults is well established, but in the pediatric population, stroke management guidelines are still being developed. The total number of strokes in children treated with thrombectomy to date is unknown. For this reason, the data on complications of this type of treatment are probably underestimated in the literature. The safety and efficacy of mechanical thrombectomy in children are still unknown. Therefore, registries of children treated in this way should be created, and existing ones, such as the International Pediatric Stroke Study Registry, should be used. Previous publications indicate the effectiveness of recanalization and improvement of the patients’ neurological condition at the level of 89.5% (46). In another study, 83% of thrombectomies achieved recanalization of modified thrombolysis in cerebral infarction (mTICI) ≥2b (47). However, the study groups are still too small—19 and 21 patients, respectively. Further research on the effectiveness of thrombectomy is vital. Specific differences between children and adults in performing mechanical thrombectomy are among others: smaller diameter of the vessels (femoral and cerebral arteries), limitations in the supply of radiological contrast due to the lower body weight of children, exposure to X-ray radiation in young children, arteriopathy that causes AIS in children (there are concerns about inserting a catheter into an acutely inflamed artery in focal cerebral arteriopathy or chronically narrowed cerebral arteries in moyamoya disease) (10). In children, the initial PedNIHSS score directly predicts prognosis; consequently, the risk of thrombectomy may outweigh the benefits in children with a low PedNIHSS score. Therefore, thrombectomy is considered in children ≥6 PedNIHSS (48). According to the American Heart Association/American Stroke Association (AHA/ASA) statement it would be reasonable to perform thrombectomy in children who meet the following criteria: persistent neurological deficits ≥6 PedNIHSS, radiological confirmation of large cerebral artery occlusion, use of the method in larger children because of the fear of catheter insertion into small femoral and cerebral arteries, and restrictions on contrast administration and radiation exposure, treatment decision made together with neurologists experienced in the treatment of stroke in children, an intervention undertaken by a neuroradiologist experienced in performing thrombectomy in both children and adults (10). The patient in the described case underwent a thrombectomy because she was disqualified from intravenous thrombolysis. Still, she also met the aforementioned criteria—her PedNIHSS score was 18 points, angiography showed an occlusion of one of the branches of the right middle cerebral artery in the M2 segment, and the decision to intervene was made based on a multidisciplinary consultation.

Attention should also be paid to the organizational problem regarding the treatment of strokes in children. In the described case, the patient was transferred from a pediatric hospital to an adult hospital for thrombectomy and recovery. After the procedure, she was taken to the anesthesiology and intensive care ward of a children’s hospital. Mechanical thrombectomy was performed 3 h and 15 min after the onset of symptoms as a result of efficient cooperation between the centers. For this reason, it is worth developing a network of specialists from different fields, located in several hospitals, as well as guidelines for treating AIS in children to optimize patient safety. Due to the growing scale of the problem, it may be necessary to establish stroke centers for children in the future. The use of thrombectomy in the pediatric population may also significantly reduce the cost of medical care in the future because it could prevent disability and eventually rehabilitation and other treatment after stroke’s consequences (10).

## 4 Conclusion

Thrombectomy method can be used in the treatment of ischemic stroke in children to reduce mortality, improve quality of life, and prevent disability. Guidelines are needed for using the thrombectomy method in children. Until then, a multidisciplinary team of pediatric neurologists and interventional radiologists should assess patients for this procedure. The occurrence of an episode of cerebral thrombosis in a child should prompt clinicians to look for risk factors for ischemic stroke in a patient, which include antiphospholipid syndrome, among others.

## Data Availability

The original contributions presented in the study are included in the article/supplementary material, further inquiries can be directed to the corresponding author.
